# A novel assay provides sensitive measurement of physiologically relevant changes in albumin permeability in isolated human and rodent glomeruli

**DOI:** 10.1016/j.kint.2017.12.003

**Published:** 2018-05

**Authors:** Sara Desideri, Karen L. Onions, Yan Qiu, Raina D. Ramnath, Matthew J. Butler, Christopher R. Neal, Matthew L.R. King, Andrew E. Salmon, Moin A. Saleem, Gavin I. Welsh, C. Charles Michel, Simon C. Satchell, Andrew H.J. Salmon, Rebecca R. Foster

**Affiliations:** 1 Bristol Renal, Bristol Medical School, University of Bristol, Bristol, UK; 2 Department of Bioengineering, Imperial College, London, UK

**Keywords:** albuminuria, angiopoietin-1, diabetes, endothelial glycocalyx, glomerular permeability, steroid-resistant nephrotic syndrome plasma

## Abstract

Increased urinary albumin excretion is a key feature of glomerular disease but has limitations as a measure of glomerular permeability. Here we describe a novel assay to measure the apparent albumin permeability of single capillaries in glomeruli, isolated from perfused kidneys cleared of red blood cells. The rate of decline of the albumin concentration within the capillary lumen was quantified using confocal microscopy and used to calculate apparent permeability. The assay was extensively validated and provided robust, reproducible estimates of glomerular albumin permeability. These values were comparable with previous *in vivo* data, showing this assay could be applied to human as well as rodent glomeruli. To confirm this, we showed that targeted endothelial glycocalyx disruption resulted in increased glomerular albumin permeability in mice. Furthermore, incubation with plasma from patients with post-transplant recurrence of nephrotic syndrome increased albumin permeability in rat glomeruli compared to remission plasma. Finally, in glomeruli isolated from rats with early diabetes there was a significant increase in albumin permeability and loss of endothelial glycocalyx, both of which were ameliorated by angiopoietin-1. Thus, a glomerular permeability assay, producing physiologically relevant values with sufficient sensitivity to measure changes in glomerular permeability and independent of tubular function, was developed and validated. This assay significantly advances the ability to study biology and disease in rodent and human glomeruli.

The ability to measure glomerular permeability is essential in the detection and assessment of glomerular disease. Albuminuria is the standard measure; however, it does not accurately reflect glomerular permeability. This is due to confounding changes in local hemodynamics, as well as tubular reabsorption of filtered albumin.^1^ For experimental studies of human glomerular permeability, measurements of albuminuria are not possible. Therefore, the development of a reliable, physiologically relevant assay to directly measure glomerular permeability using isolated glomeruli would be very useful and independent of confounding variables. We have previously developed a reliable assay of water permeability in isolated glomeruli^2^ and here we describe the development of an albumin permeability assay. In 1992, Savin *et al.* modified an isolated glomerular oncometric assay, originally developed to assess hydraulic conductivity,^3^ to measure glomerular reflection coefficients^4^ and further modified the assay in 2015 for higher thoughput.^5^ However, this assay is limited to measuring relative changes in permeability. Daniels *et al.* developed an *ex vivo* method to quantify the apparent diffusion of fluorophores across individual glomerular capillaries in cross-section,^6^ yet permeability values were significantly higher than values previously measured *in vivo*.^7^ Using similar principles we developed an assay to measure apparent, diffusive glomerular albumin permeability in isolated glomeruli deriving physiologically relevant values.

We aimed to develop and characterize an assay that could sensitively measure glomerular albumin permeability and demonstrate its utility under different pathophysiological conditions, in different species. The pathophysiological variables tested were: (i) disruption of endothelial glycocalyx,^8^, ^9^, ^10^ (ii) exposure to plasma from patients with recurrence of nephotic syndrome posttransplant,^11^, ^12^ (iii) early diabetes,^13^, ^14^ and (iv) rescue of glomerular permeability in diabetes using angiopoietin-1.^15^, ^16^

## Results

### Set-up and characterization of a novel glomerular albumin permeability assay

**Figure 1:**
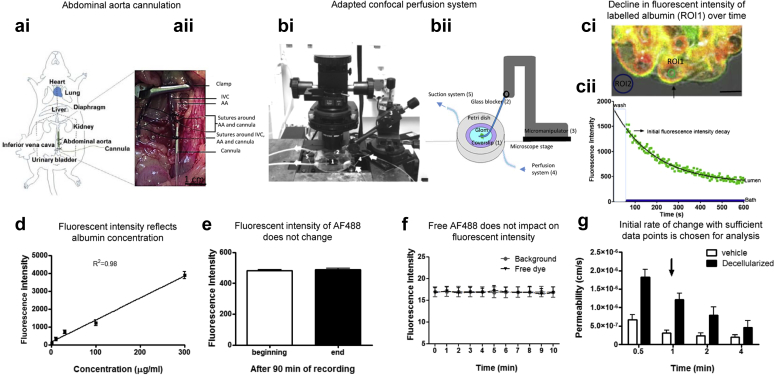
**Glomerular albumin permeability assay setup and characterization.** (**a**) A midline laparotomy was used to expose the abdominal aorta, which was cannulated with a blunted needle, as detailed in (**i**) and (**ii**). AA, abdominal aorta; IVC, inferior vena cava. (**bi**) The photograph shows an inverted confocal microscope equipped with a modified Petri dish consisting of a central hole with a glass coverslip (0.085-mm thickness) attached on the top and connected to a perfusion and a suction system. Glomeruli were trapped on the (1) glass coverslip by a (2) glass restrainer positioned using a (3) micromanipulator. Ringer bovine serum albumin (BSA) was pumped into the Petri dish though a (4) perfusion system and simultaneously removed though a (5) suction system to wash the glomeruli of surrounding Alexa Fluor 488 (AF488) BSA (20–60 seconds). Arrows indicate the direction of flow. A schematic is shown to reflect the setup showing a glomerulus trapped by a micromanipulator connected to a restraining glass pipette. (**bii**) Numbers relate to those shown in (**bi**). (**ci**) An image of peripheral capillary loops of a trapped glomerulus is shown. The capillary wall is labeled red with R18, and AF488-BSA fills the lumen (labeled yellow where there is colocalization). The arrow indicates a spherical capillary, chosen for analysis. Region of interest (ROI) 1 and ROI2 are shown in the capillary lumen and bath, respectively (bar = 5 μM). (**cii**) The decline in fluorescence intensity of labeled albumin over time is plotted. The green boxes represent the flux of movement of fluorescently labeled albumin molecules from the lumen to the bath (ROI1), and the blue line represents the fluorescence intensity of the bath after the wash (ROI2). The decline in fluorescence intensity follows a single exponential decay (green boxes, r^2^ = 0.97). (**d**) Mean fluorescence intensity was plotted against AF488-BSA concentrations. (**e**) The initial fluorescence intensity values were compared with the FI values at the end of the experiment (after 90 minutes of laser exposure; paired *t* test, *P* = 0.20). Free dye fluorescence intensity is plotted against time and is not different than background. (**f**) Permeability between decellularized and normal (vehicle-treated) glomeruli were analyzed for different periods of time following the washout period. (**g**) The period chosen for analysis after the washout period was 1 minute (arrow) because this had a significant number of frames to analyze (30 seconds was too few) and differentiated well between treatment groups. To optimize viewing of this image, please see the online version of this article at www.kidney-international.org.

The abdominal aorta of rats and mice were cannulated to perfuse both kidneys (Figure 1a). Kidneys were perfused first with mammalian 4% Ringer bovine serum albumin (BSA) to flush out red blood cells (which interfere with analysis). They were then perfused with octadecyl rhodamine B chloride (R18, a lipophilic membrane stain) in 4% Ringer BSA, to label cell membranes red in order to distinguish the glomerular capillary walls. This was followed by perfusion with Alexa Fluor 488 (AF488; Thermo Fisher Scientific, Waltham, MA) labeled 4% BSA. Perfused glomeruli were isolated by sieving, then incubated in AF488-BSA with or without treatments. A single glomerulus was then placed in an adapted petri dish (Figure 1b) on a confocal microscope in AF488-BSA. This was trapped to micro-occlude the afferent and efferent arterioles using a glass micropipette, thereby sealing the glomerulus so that any movement of molecules could only occur across the capillary walls by diffusion. Regions of interest (ROI) were chosen within circular capillary loops (ROI1) and external to the glomerulus (ROI2, Figure 1ci). The 4% AF488-BSA was exchanged for iso-oncotic unlabeled 4% BSA, and the initial change in fluorescence intensity was measured over time in both ROI (Figure 1cii). The fluorescence intensity and concentration of AF488-BSA were linearly related (Figure 1d), the fluorescence intensity of AF488 was not impacted by time (Figure 1e), and AF488 free dye at 0.07% or below had no impact on fluorescence intensity (Figure 1f). The decline in fluorescence intensity in ROI1 was measured over 1 minute, which was the minimum length of time necessary to give sufficient data points to represent the initial decline (Figure 1g).

To demonstrate that macromolecules were effectively restricted by glomeruli in this assay, 2 different molecular weight dextrans conjugated with fluorescein isothiocyanate (FITC) were used. There was a linear relationship between low molecular weight (LMW) and high molecular weight (HMW) dextran concentration and fluorescence intensity (Supplementary Figure S1A). Isolated glomeruli were significantly more permeable to LMW FITC-dextran (4 kDa) compared with HMW FITC-dextran (500 kDa), as predicted (Supplementary Figure S1B). However, baseline values for FITC-500 kDa dextran were much higher than anticipated. This is likely due to the overestimation of permeability of dextran due to the rapid photobleaching of FITC in our system (Supplementary Figure S1Ci and Cii). Although these data suggest that FITC is inferior to AF488 in our assay, the data nevertheless confirm lower glomerular permeability to a higher–molecular weight tracer.

### The glomerular permeability assay accurately and reproducibly measures albumin permeability

**Figure 2:**
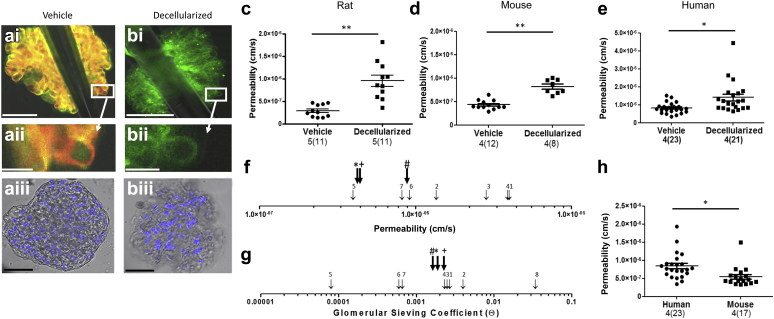
**The glomerular albumin permeability assay demonstrates physiologically relevant values in rodents and humans.** (**a–c,f**) Rats and (**d**) mice (FVB, mixed background) were perfused with Alexa Fluor 488 bovine serum albumin (AF488-BSA) (green) and R18 (red). (**e,h**) Human unused donor kidneys were perfused, and glomeruli were isolated and then incubated sequentially in fluorophores. Representative images of (**a**) vehicle-treated and (**b**) decellularized glomeruli are shown. (**i**) shows a fluorescent image of a whole glomerulus trapped by a glass pipette, (**ii**) shows a higher magnification of (**i**) as indicated, and (**iii**) shows representative phase contrast/fluorescent images of the whole glomeruli with Höescht 33342 staining. These demonstrate (**i, ii**) the loss of R18 and (**iii**) the partial loss of Höechst 33342 (blue) in treated glomeruli. Bar = 50 μm (**i, iii**) or 5 μM (**ii**). (**c,e**) Apparent glomerular albumin permeability (Ps’alb) was measured (unpaired *t* tests). (**f**) Ps’alb values from our glomerular permeability assay (bold black arrows, # = human Ps’alb, * = rat Ps’alb, and + = mouse Ps’alb) were compared with those previously reported and shown on a logarithmic scale (citation numbers refer to references described in Table 1). (**g**) Glomerular sieving coefficients were calculated from Ps’alb (shown in Table 2) and compared with previously reported values (bold black arrows, # = human,* = rat, + = mouse), shown on a logarithmic scale (for details see Supplementary Methods; citation numbers refer to references described in Table 3). (**h**) To compare human Ps’alb values more directly with experimental values, aged mouse (C57/BL6, 1-year-old) kidneys were perfused then incubated in fluorophores, and Ps’alb was measured (unpaired *t* test). Data for Ps’alb assays are presented as glomerular averages, with the number of glomeruli in parentheses and the number of animals or humans before the parentheses. Statistical analyses were performed on the number of animals per group. Bonfferoni *post hoc* tests were used except with comparisons of human data, which were not normally distributed and therefore with which Mann-Whitney *post hoc* tests were used. *P < 0.05, **P < 0.01. To optimize viewing of this image, please see the online version of this article at www.kidney-international.org.

The apparent glomerular albumin permeability (Ps’alb) assay was used to demonstrate that physiologically relevant permeability values could be measured in glomeruli from different species, including humans. The cellular components of the glomerular filtration barrier contribute substantially to macromolecular permeability control.^6^ Therefore intact rodent and human glomeruli were compared with partially decellularized glomeruli to demonstrate that the assay could measure changes in glomerular permeability associated with loss of the cellular components in a similar manner to that used by Daniel’s *et al.*^6^ Confocal fluorescence imaging confirmed removal of cell membranes from glomeruli (by loss of R18 staining) although there was some residual nuclear material (Figure 2a and b). Ps’alb values were significantly higher for partially decellularized glomeruli than intact glomeruli in all species examined (Figure 2c–e). Human, rat, and mouse Ps’alb values were all of a similar magnitude to those previously reported in systemic vessels *in vivo* (and *ex vivo*) (Figure 2f, Table 1). Glomerular permeability is more often measured by glomerular sieving coefficients (GSC) *in vivo*. To understand how Ps’alb values related to previously published GSC values in rat, mouse and human glomeruli, the membrane pore model of permeability was used to convert Ps’alb to equivalent values of GSC (Table 2) (see “Calculation of GSC values” in Supplementary Methods for details). Human-, rat-, and mouse-converted GSC values (0.00182, 0.0027, and 0.002, respectively) were very similar to those previously reported (Figure 2g, Table 3). Importantly, we have shown for the first time that human Ps’alb can be measured directly and with physiologically relevant values. Methodology varied between rodent and human glomeruli preparations, because it was impractical to perfuse whole human kidneys with fluorophores, although all kidneys were perfused with a physiological salt solution. There was also a 24-hour time delay in processing human kidneys. In 1 set of experiments, rat and mouse glomeruli were therefore also incubated *ex vivo* with fluorophores in the same way as human glomeruli. Glomeruli treated in this way still had significantly lower permeability than the human glomeruli, confirming that the different methods for applying fluorophores did not account for species differences. Furthermore, there were no significant differences in Ps’alb between these 2 methodologies in either rats or mice (Supplementary Figure S2A and B). Glomeruli that were isolated for up to 4 hours before measurements did not have significantly different Ps’alb, suggesting that up until this point glomerular permeability is stable (Supplementary Figure S2C). All our previous observations on rodent glomeruli were performed within this time frame. In another set of experiments, mouse kidneys were kept on ice for 24 hours before glomeruli were isolated to compare with the delayed processing time frame of human kidneys. There was a significant increase in Ps’alb in mouse glomeruli processed after 24 hours (Supplementary Figure 2D), indicating that the prolonged cold storage of human organs inherent in the retrieval process may provide some of the explanation for higher permeability values. Ps’alb was significantly higher for human glomeruli than these mouse glomeruli, indicating a species difference or other residual differences (Figure 2h). Of note, mice in these experiments were aged to 1 year to relate to aged human kidneys (from donors 72 ± 3.1 years of age).

**Table 1:** Ps’alb values in previously described models

	Species	Method	Ps’alb value	Study
1	Frog	*In vivo* mesenteric vessel cannulation	4e-006 cm/s (15 cm H_2_O capillary pressure)	Huxely *et al.*, 1987^7^
2	Rat	*Ex vivo* isolated glomeruli	1.36e-0.006 cm/s	Daniels *et al.*, 1993^6^
3 4 5	Pig	*Ex vivo*, coronary microvessel, isolated and cannulated	2.85e-006 cm/s 3.92e-006 cm/s (11 cm H_2_O capillary pressure) 4.0e-007cm/s (15.3 cm H_2_O arteriole pressure)	Yuan *et al.*, 2000^46^ Yuan *et al.*, 1993^47^ Huxley *et al.*, 2000^48^
6	Rat	*In vivo* venular microvessels	0.92e-006 cm/s (20 cm H_2_O microvessel pressure)	Adamson *et al.*, 2014^49^
7	Rat	*In vivo* mesenteric vessel cannulation	0.82e-006 cm/s	Cai *et al.*, 1985^50^

Ps’alb, apparent glomerular albumin permeability.

**Table 2:** GSC converted from Ps’alb for human rat and mouse glomeruli

Coefficient	Mouse	Rat	Human
P albumin (cm/s)	4.3 x 10^–7^	4.5 x 10^–7^	8.942 x 10^–7^
Lp cm/(sec dyne) x 10^–8^	2.1	1.629	2.735
Single pore population, pore radius, R (nm)	3.742	3.69	3.776
Single pore: θ (GSC)	0.0026	0.00068	0.0016
Two-pore population: R_L_ = 11 nm,^27^ Equivalent θ, (GSC)	0.002	0.0027	0.00182

GSC, glomerular sieving coefficient; Ps’alb, apparent glomerular albumin permeability.

See explanation in Supplementary Materials and Methods.

**Table 3:** GSC values in previously described models

	Species	Method	Ps’alb	Study
1 2	Rat	*In vivo*, confocal, glomerular	0.0027 0.0004	Stolte *et al.*, 1979^51^ Tanner *et al.*, 2009^52^
3	Mouse	*In vivo,* multiphoton imaging, glomerular	0.0025	Nakano *et al.*, 2012^53^
4	Mouse	Cooled isolated perfused kidney	0.0023	Jeansson *et al.*, 2003^8^
5	Human	Fanconi syndrome, HPLC, and MALDI-TOF MS urine analysis	0.00008	Norden *et al.*, 2004^34^
6	Rat	Fractional micropuncture	0.0006	Tojo and Endou, 1992^54^
7	Rat	Labeled normal and neutral albumin	0.0006	Bertolatus and Hunsicker, 1985^55^
8	Rat	Intravital 2-photon microscopy	0.034	Russo *et al.*, 2007^56^

HPLC, high-performance liquid chromatography; MALDI-TOF MS, Matrix-assisted laser desorption ionization-time of flight mass spectrometry; Ps’alb, apparent glomerular albumin permeability.

### Utility of the glomerular albumin permeability assay

We took a number of approaches to test that the glomerular Ps’alb assay could effectively measure changes in glomerular macromolecular permeability.

#### Endothelial glycocalyx (eGLX) dysfunction

**Figure 3:**
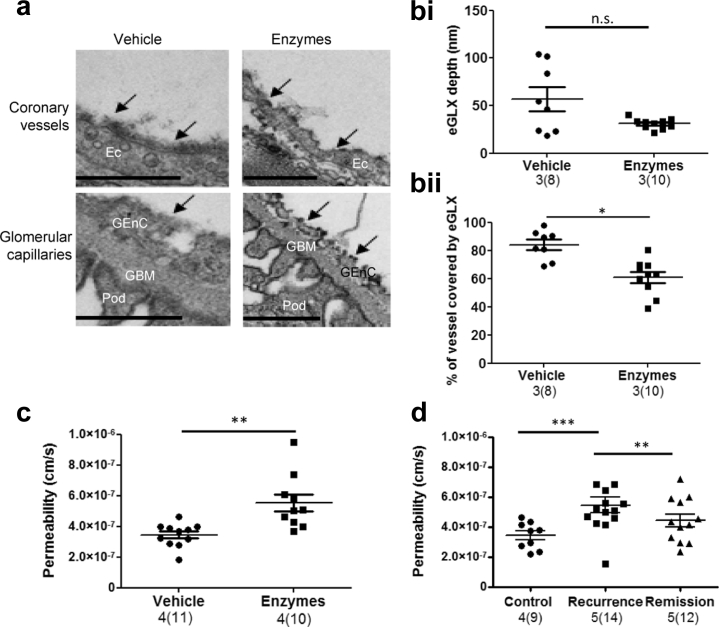
**The glomerular albumin permeability assay can be used to examine the contribution of endothelial glycocalyx (eGLX) dysfunction and human steroid-resistant nephrotic syndrome (SRNS) recurrence plasma to glomerular permeability.** Glomeruli were isolated from mice treated with glycosaminoglycan enzymes or vehicle to induce systemic eGLX disruption. (**a**) Mice were perfusion-fixed in the presence of Alcian blue, and representative electron micrographs of eGLX (black arrows) are shown for coronary and glomerular microvessels (bar = 500 nm). (**b**) Quantification of (**i**) eGLX depth and (**ii**) vessel coverage by eGLX is shown (unpaired *t* test). (**c**) Under the same conditions, mice were perfused with R18 then Alexa Fluor 488 bovine serum albumin (AF488-BSA), glomeruli were isolated, and apparent glomerular albumin permeability (Ps’alb) was measured (unpaired *t* test). Rat kidneys were perfused with R18 and AF488-BSA, then glomeruli were isolated and incubated in 10% human SRNS recurrence or remission plasma or control (Ringer BSA) for 1 hour at 37 °C. (**d**) Ps’alb was measured (1-way analysis of variance with Bonferroni *post hoc* tests indicated). Number of rats used, *n* = 5; number of patient plasma pairs, *n* = 4. Total number of glomeruli shown. Data for Ps’alb assay are presented as glomerular averages with the number of glomeruli in parentheses and the number of animals before the parentheses. Statistical analyses were performed on the number of animals per group. *P < 0.05, **P < 0.01, *** *P* < 0.001. EC, endothelial cells; GBM, glomerular basement membrane; GEnC, glomerular endothelial cells; Pod, podocyte foot processes. To optimize viewing of this image, please see the online version of this article at www.kidney-international.org.

eGLX is situated on the luminal side of glomerular and other endothelial cells^17^, ^18^ and is the first (most “upstream”) component of the glomerular filtration barrier to encounter albumin.^19^ Damage to eGLX decreases glomerular filtration barrier albumin permselectivity as demonstrated by fractional clearance of labeled Ficoll and albumin in cooled isolated perfused mouse kidneys.^8^, ^9^ To test the physiological sensitivity of the assay, eGLX dysfunction was induced with an i.v. bolus of hyaluronidase and chondroitinase 30 minutes before killing. Representative electron micrographs are shown to demonstrate systemic eGLX removal by GAG-degrading enzymes in coronary and glomerular microvessels (Figure 3a). While eGLX depth was not significantly decreased, vessel coverage by eGLX was (Figure 3b), suggesting a “patchy” coverage as demonstrated in the micrographs. Of note, we have previously shown that both vessel coverage by eGLX and eGLX depth are inversely related to permeability.^20^ The enzymes induced a significant increase in Ps’alb as anticipated (Figure 3c). No significant differences were found in podocyte foot process width, foot process length, number of foot processes per μm GBM, slit diaphragm length, number of endothelial fenestration per μm GBM, and GBM width (data not shown), suggesting that effects of these enzymes on the glomerular filtration barrier are limited to the eGLX.

#### Human steroid-resistant nephrotic syndrome (SRNS) plasma

To test the use of clinical samples on this assay, plasma was used from individuals with SRNS that had undergone plasma exchange during periods of posttransplant recurrence, or subsequent remission, with the assumption being that in recurrence the “permeability factors” were present and in remission they were not.^21^ Rats were perfused with R18 and AF488-BSA, and glomeruli were isolated and incubated in 10% plasma from SRNS patients for 1 hour. SRNS recurrence plasma significantly increased Ps’alb in rat glomeruli compared with remission plasma, which was very similar to control conditions (no plasma) (Figure 3d).

### The Ps’alb assay is sufficiently sensitive to measure early glomerular damage

**Figure 4:**
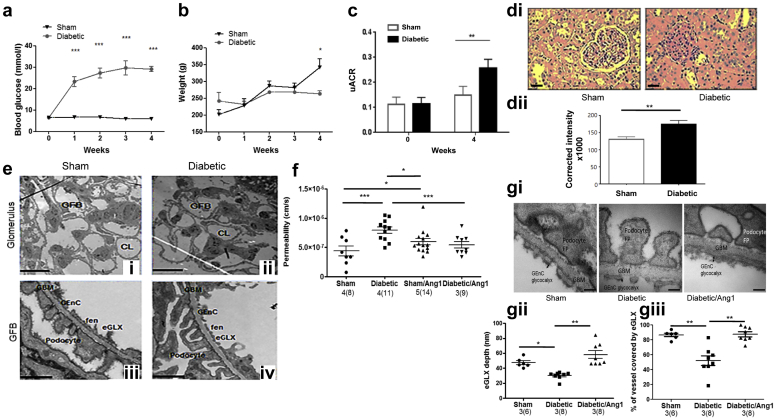
**The glomerular albumin permeability assay is sufficiently sensitive to measure diabetes-induced changes associated with mild kidney dysfunction.** (**a**) Blood glucose (*n* = 4) and (**b**) body weight (*n* = 6) were measured weekly before and after the induction of diabetes in rats (2-way analysis of variance, Bonferroni *post hoc* indicated on the graph). (**c**) Urinary albumin-to-creatinine ratios (uACR) were measured at week 0 and week 4 in control (sham) and diabetic rats (*n* = 6, unpaired *t* test). Periodic acid–Schiff staining was (**di**) imaged (bar = 25μm) and (**dii**) quantified between control and diabetic rats 4 weeks after streptozotocin (STZ) was administered (unpaired *t* test, *n* = 4). Diabetic (**ii, iv**) and control rats (**i, iii**) were perfusion-fixed 4 weeks after STZ, and kidneys were processed and imaged by transmission electron microscopy. (**e**) Representative images of the glomerulus (**i, ii**; bar = 10 μm) and higher magnifications of the glomerular filtration barrier (GFB; **iii, iv**; bar = 500 nm) are shown. (**f**) Glomeruli isolated from control or diabetic rats at 4 weeks were incubated in vehicle or Ang-1 (200 ng/ml) for 1 hour, and apparent glomerular albumin permeability was measured (1-way analysis of variance, Bonferroni *post hoc* tests indicated on the graph). Data are presented as glomerular averages with the number of glomeruli in parentheses and the number of animals before the parentheses. Statistical analyses were performed on the number of animals. Diabetic and control rats were given Ang1 i.p. 30 minutes before killing. They were then whole-body perfusion-fixed in the presence of Alcian blue. (**gi**) Representative transmission electron micrographs are shown (bar = 100 nm). (**gii**) EGLX depth and (**giii**) coverage were quantified (1-way analysis of variance with Bonferroni *post hoc* tests indicated). *P < 0.05, **P < 0.01, *** *P* < 0.001. CL, capillary loop; eGLX, endothelial glycocalyx; fen, fenestrations; FP, foot processes; GBM, glomerular basement membrane; GEnC, glomerular endothelial cells; GFB, glomerular filtration barrier. To optimize viewing of this image, please see the online version of this article at www.kidney-international.org.

To further investigate the sensitivity of the Ps’alb assay, a rat model of early glomerular damage was utilized. Rats given streptozotocin (STZ, a type 1 model of diabetes) were significantly hyperglycemic at 1 week after injection, which was sustained (Figure 4a), confirming diabetes. Weight gain in diabetic rats was significantly reduced as anticipated (Figure 4b). Diabetic rats showed a modest, but significant, increase in urinary albumin-to-creatinine ratio (1.7-fold increase) compared with control rats 4 weeks after injection (Figure 4c), suggesting a modest increase in glomerular leak of albumin. It is important to note that albuminuria is independent of STZ-induced renal toxicity in this rat model (although total proteinuria is not).^22^ Mesangial matrix expansion was assessed using periodic acid–Schiff staining, demonstrating a mild but significant increase in glomerular fibrosis in diabetic rats (Figure 4di and ii). Electron microscopy was used to analyze glomerular ultrastructure. No significant changes were observed (representative images Figure 4ei–iv, data not shown). Together these data confirm that these diabetic rats have early signs of glomerular damage rather than established diabetic nephropathy. Diabetes induced a significant increase in Ps’alb, which was ameliorated by the paracrine growth factor, angiopoietin-1 (Ang1), to a level comparable with nondiabetic glomeruli (Figure 4f), supporting previously published results.^15^ Ang1 alone had no effect compared with sham, suggesting no effect on otherwise healthy capillaries under nondiabetic conditions. eGLX is damaged in models of diabetes.^23^ We have previously shown that Ang1 acts on glomerular endothelial cells^24^ and that it can increase eGLX depth and reduce microvascular permeability in a different capillary bed.^16^ Therefore, we investigated whether the protective effect of Ang1 was associated with restoration of eGLX damage. Glomerular eGLX depth and coverage was significantly reduced in diabetic rats compared with control rats, but was restored by Ang1 (Figure 4gi–iii). Together these data confirm that the Ps’alb assay can sensitively measure early changes in glomerular barrier function associated with eGLX dysfunction.

## Discussion

We have developed a glomerular albumin permeability assay that gives physiologically relevant permeability values and allows human Ps’alb measurements for the first time. We demonstrated utility of the assay and confirmed that it was sufficiently sensitive to measure early decline in glomerular permeability and could be used in conjunction with clinical samples.

Ps’alb measurements were reassuringly close to those measured previously *in vivo*^7^, ^8^ and even lower than previously measured Ps’alb values *ex vivo.*^6^ This demonstrates the strength of using permeability values compared with using relative changes in permeability, as used previously.^4^ It allows us to directly compare with previously reported values and to interpret our data in the context of health and disease. Knowing that the permeability of glomeruli are within the physiological range confirms the relevance of our observations to the *in vivo* situation.

Typically, previously published values of glomerular albumin permeability are presented as GSC. These reflect the fraction of plasma albumin that can pass though the glomerular filtration barrier, expressed as values over the range of 0 to 1, with 1 being free passage of albumin. Theories of glomerular capillary permeability to fluid and albumin are of 3 general categories: (i) pore theories,^25^, ^26^, ^27^ (ii) fiber-matrix theories,^28^, ^29^, ^30^, ^31^ and (iii) structural theories, which model diffusion and flow through geometric simplifications of pathways proposed for water and albumin from tissue ultrastructure.^32^ We have used pore theory, not because we believe the pathways between capillary lumen and Bowman’s space are cylindrical pores, but because it is an internally consistent mathematical model that allows one to estimate the GSC from the diffusional permeability coefficients and glomerular hydraulic conductivity, using the minimum number of assumptions. The fiber-matrix theories, while more realistic in terms of representing the molecular sieving processes through the eGLX and the GBM, are able to describe diffusion of solutes through a matrix of cylindrical fibers of a given radius and occupying a known fraction of the matrix, but calculation of flow and solvent drag of solutes through the matrix require additional assumptions including the orientation of the fibrous molecules with respect to the flow.^33^ Furthermore, pore theory has been used to interpret the GSC for albumin and a range of neutrally charged proteins in rats,^27^ and this allows us to compare our calculated estimates of GSC from rat glomerular capillary P’alb coefficients with measured values of GSC in the rat. Like Lund *et al.*^27^ we assumed the pathways between glomerular capillary lumen to consist of 2 populations of pores: a very large population of “small pores” that accounted for the flow and diffusion of nearly all the water and small hydrophilic solutes, and a very small population of large pores through which most of the albumin would be filtered. The calculations of GSC from our measurements of Ps’alb and published values for glomerular capillary hydraulic conductivity are given in the Supplementary Material (see “Calculation of GSC values”). In brief, analysis of our measured values of Ps’alb gave a maximum value for the radius of the small pore population and, combining this with measured glomerular capillary hydraulic conductivity values and the value assumed by Lund *et al.*^27^ for the radii of the large pores, allowed us to estimate the fractions of water flow through small and large pore populations and the GSC. The calculated values of GSC for rats, mice, and humans (0.00182, 0.0027, and 0.002, respectively) were very similar to *in vivo* rat values and isolated cooled mouse kidney values.^8^ GSC were estimated previously in isolated rat glomeruli giving values of 0.13 ± 0.02,^5^ 2 orders of magnitude greater than our and other reported values. We have shown the first direct measurements of human Ps’alb, from which we calculated GSC. These were higher than those measured from patients with Fanconi syndrome (0.00008, the only other human GSC value). However, that published value assumed that tubular reabsorption is completely abolished (which may not be the case)^34^ and is very low even compared with other *in vivo* values measured directly. Of course, we have not measured GSC directly, and we recognize the limitations of using pore theory to calculate these.

Our work confirms that targeted disruption of eGLX significantly changes Ps’alb. Previous work has demonstrated albumin leak (by immunogold) across the GFB due to eGLX dysfunction over a period of 4 weeks.^10^ Increased Ps’alb was measured in glomeruli isolated from diabetic rats, even in those with mild proteinuria, suggesting that the assay is sensitive to early changes in renal function Albuminuria can be impacted by tubular reuptake of albumin resulting in variable urinary albumin-to-creatinine ratio values in experimental diabetic models.^35^ When tubular reuptake of albumin was inhibited using an inducible transgenic megalin knockout mouse line, this resulted in a 10-fold increase in urinary albumin secretion in diabetic mice.^36^ Because our Ps’alb assay measures albumin permeability directly, it circumvents this potential confounding factor and confirms that glomeruli are a target in diabetic kidney disease.

eGLX dysfunction is associated with increased vascular permeability,^37^, ^38^ including albuminuria, indicating dysfunction of the glomerular filtration barrier.^13^, ^14^, ^39^ Albuminuria is also associated with reduced eGLX dimensions^40^ including in patients with type 1^41^ and type 2 diabetes.^14^ Here we demonstrate that Ang1 reduced elevated Ps’alb in diabetic rats, consistent with previous reports,^15^, ^42^ and also restored the diabetes-associated loss of glomerular endothelial cell GLX. Of note, because these diabetic rats had low-level proteinuria and minimal ultrastructural changes, we speculate that the increased glomerular albumin permeability was largely due to eGLX dysfunction.

Importantly, this is the first demonstration that Ang1 could restore eGLX in glomeruli. In diabetic mice where Ang1 was overexpressed in a podocyte-dependent manner, albuminuria was significantly reduced and endothelial cells were shown to be protected. However, endothelial ultrastructure, including the eGLX, was not studied in detail.^15^ Ang1 depletion in a type 1 model of diabetes substantially increased albuminuria^42^ supporting the important role of Ang1 in renal protection. We have previously shown that Ang1 can protect against vascular endothelial barrier dysfunction though modification of the eGLX *in vivo,*^16^ but this study confirms for the first time that Ang-1 also rapidly restores the eGLX (within 30 minutes).

Not only have we demonstrated that this assay can be applied to human glomeruli, but we have also demonstrated that it can be used in conjunction with human clinical samples after nephrectomy. There is a continuing interest in the field to elucidate the mechanism behind circulating permeability factors in SRNS patients and how they differ between states of recurrence and remission.^12^, ^43^, ^44^ We have confirmed previous findings, that plasma from SRNS patients in recurrence causes a significant increase in Ps’alb, whereas remission does not.^11^ This demonstrates proof of principle that, not only can we compare apparent Ps’alb in response to clinical samples, but that human clinical samples could be used to determine mechanism in *human* glomeruli, which is an important leap forward for the field.

In summary, we have developed and characterized a novel glomerular permeability assay that can measure early decline in glomerular permeability. This represents a new gold standard in *ex vivo* glomerular permeability measurement, and is an important research tool that could be used in experimental models and, critically, in human clinical samples to determine human glomerular permeability.

## Concise Methods

All materials were purchased from Sigma-Aldrich (Dorset, UK) unless otherwise stated.

All animal experiments performed were in accordance with UK Home Office regulations. All studies on human kidney tissue and human SRNS plasma were approved by national and local research ethics committees (Institutional Ethical Committee, Leiden University Medical Centre, The Netherlands; South West–Central Bristol National Health Service Research Ethics Committee, UK; and East Midlands–Leicester National Health Service Research Ethics Committee, UK), and conducted in accordance with the tenets of the Declaration of Helsinki.

### Rat glomeruli isolation and treatment

Adult male Sprague Dawley rats (Harlan Laboratories, UK) aged 6 to 8 weeks (200–380 g) (or 4 weeks after STZ) were anesthetized with 3.5% isoflurane and sodium pentobarbital 60 mg/ml given at 1 μl/g of body weight. Once stable anesthesia had been achieved, the animal was laid on its back. The abdominal aorta was then exposed with a midline laparotomy and a cannula consisting of a 23-gauge, 1-inch blunted needle (Terumo, UK) attached to thin laboratory tube (0.58 × 0.19 mm; TUB3662, Scientific Laboratory Supplies, UK), and it was placed in the infrarenal abdominal aorta (Figure 1ai and ii). A clamp was applied above the branch point of the right kidney, and a suture was pulled around the vena cava and abdominal aorta below the cannulation site to maximize perfusion of the left kidney. Both kidneys were perfused with 4% mammalian Ringer BSA solution (NaCl, 132 mM; KCl, 4.6 mM; MgSO^4-^7H_2_O, 1.27 mM; CaCl_2_-2H_2_O, 2 mM; NaHCO_3_, 25 mM; D[+]glucose, 5.5 mM; N-2-hydroxyethylpiperazine-N’-2-ethanesulphonic [HEPES] acid, 3.07 mM; HEPES sodium salt, 1.9 mM; 4% BSA, pH 7.40 ± 0.01 using 115 mM HCl or NaOH to maintain osmolarity) containing R18, (36.5 μg/ml, Thermo Fisher Scientific O-246) followed by AF488-conjugated BSA (AF488-BSA, 30 μg/ml). Following exsanguination and nephrectomy, glomeruli were isolated on ice by graded sieving from the cortex of each kidney as previously described, with the glomeruli collected from the 125 μm–gauge sieve.^3^ Glomeruli were incubated in AF488-BSA on ice until use.

### Mouse glomeruli isolation

Mice (including FVB/mixed background, bred in-house, male db/+ lean mice age 12–14 weeks from the Jackson Laboratory, and adult female C57BL/6 mice, 37–27 g from Charles River) were anesthetized using an i.p. injection of 200 to 800 μl of tribromoethanol (133 μg/ml in 0.9% saline). The abdominal aorta was exposed and cannulated with PE-10 tubing (427400; Becton Dickinson, Franklin Lakes, NJ), and then the protocol was followed as for rats, except that glomeruli were collected from the 75-μm sieve.

### Human glomeruli isolation

Human kidneys were perfused with physiological salt solution, as is standard practice for transplantation (which is useful for this assay because it flushes out red blood cells). Kidneys deemed unsuitable for transplantation, from individuals 72 ± 3.1 years of age, were transported to the laboratory on ice up to 24 hours after removal. Glomeruli were isolated as above and harvested from the 125-μm sieve, then centrifuged at 1200 rcf for 15 minutes to form a pellet. The next step differed from the rodent protocols; glomeruli were resuspended in R18 (36.5 μg/ml) on ice for 45 minutes, washed, and then incubated in AF488-BSA (30 μg/ml) on ice until use.

### Standardization of protocol across species

In some rats and mice the procedure was carried out as described, yet kidneys were only perfused with Ringer-BSA. Sieved glomeruli were subsequently incubated sequentially in R18, then AF488-BSA as described for human kidneys.

### Decellularization of isolated glomeruli

Isolated glomeruli (from rats, mice, and humans) were divided in 2 groups and either treated with vehicle or decellularized as described previously by Ligler and Robinson,^45^ although modifications were necessary to ensure the glomeruli maintained their structure in order to identify capillary loops. Glomeruli were lysed in N-lauroylsarcosine/Krebs (0.5%) for 45 minutes. After centrifugation (800 g for 5 minutes), they were resuspended in a 1% TitonX100 solution for 3 minutes at room temperature. Glomeruli were then centrifuged and the supernatant was removed. To remove residual gel-like nucleoprotein, glomeruli were suspended in a DNase solution (Precision HL DNase: 1μl of Precision DNase enzyme + 10X Precision DNase reaction buffer, product code: AM2222; Primer Design, UK), vortexed, and allowed to stand for 30 minutes. The glomeruli were then centrifuged, the supernatant was removed, and the resulting cell-free glomerulus preparation was rinsed twice and resuspended in 4% BSA (pH 7.4). A fraction of decellularized and vehicle-treated glomeruli were stained with Höechst 33342 (0.001%) and imaged to verify the efficacy of the treatment. Vehicle-treated and decellularized glomeruli were then incubated in AF488-BSA on ice until use.

### Calibration of the glomerular albumin permeability assay

To determine the best time frame for data analysis, the decline in fluorescence intensity in ROI1 was calculated from varying periods of time after the washout. Ps’alb values were compared between vehicle-treated and decellularized glomeruli. A calibration curve of known BSA concentrations was constructed using confocal microscopy and circular ROI. The fluorescent intensities were then plotted against concentration. To test the effects of photobleaching on AF488, several drops of AF488-BSA (30 μg/ml) were exposed to continuous 488-wavelength laser scanner for 90 minutes (*n* = 4). A comparison was also made between FITC-dextran (30 μg/ml) and AF488-dextran (30 μg/ml) exposed to continuous 488-wavelength laser scanner for 5 minutes. To determine the amount of free dye in a batch of AF488-BSA, the solution was centrifuged (3500 rpm for 30 minutes at room temperature) with Amicon Ultra-4 tubes (Ultracel 30k; Millipore, Watford, Hertfordshire, UK). Solution eluted from the column was compared with known concentrations of AF488 free dye (Life Technologies, Loughborough, UK) using a standard curve. The amount of free dye eluted from the column was then tested using the Ps’alb assay. Briefly, rat kidneys were perfused with free dye and a single glomerulus was isolated and Ps’alb measured, as detailed below for AF488-BSA.

### High and low molecular weight FITC dextran

Mouse glomeruli were prepared as described above, but the AF488-BSA was replaced with either HMW or LMW FITC-dextran (30 μg/ml, FD500S or FD4-100M, respectively). After sieving, the perfused glomeruli were incubated in the same solutions they were perfused with (HMW or LMW FITC-dextran) and stored on ice until use.

### Human SRNS plasma

Human exchange plasma samples were collected under ethical consent from 4 separate patients with SRNS that had undergone plasma exchange from periods when they were in posttransplant recurrence, or subsequent remission, as detailed previously.^21^ Rat glomeruli were isolated for the glomerular albumin permeability assay, as described above, then incubated in the presence of AF488-BSA for 1 hour at 37°C with 10% recurrence or remission plasma. Ps’alb measurements were taken directly afterwards (as described below).

### Systemic endothelial glycocalyx disruption

Chondroitinase (0.087 mU/g) and hyaluronidase (15 mU/g) in a final volume of 200 μl in saline (0.9%), or saline alone, were injected into the tail vein of anesthetized FVB/mixed background mice, bred in-house, between the ages of 10 to 12 week. Mice were maintained under anesthesia at 37°C for 30 minutes and then the protocol for either glomerular albumin permeability (below) or whole-body perfusion fixation^23^ was followed. Pilot studies revealed that 1000 fold higher concentration of each, as used by Jeansson *et al.*,^9^ did not reduce vessel eGLX coverage.

### Isolated glomerular imaging

A Nikon Tie inverted confocal microscope stage was replaced with a modified Petri dish with a thin glass coverslip (thickness: 0.085 mm; Raymond A Lam Laboratory Supplies, East Sussex, UK) attached to the top and connected to a perfusion syringe (20 ml) and a suction system (peristaltic pump; Dylade Laboratories, Fresenius, UK) (Figure 1b). The top of the microscope stage was also equipped with a micromanipulator (Scientifica, Uckfield, UK) connected to a restraining pipette. The restraining pipettes were made from borosilicate glass capillaries (1.2 mm in diameter; Harvard Apparatus, Holliston, MA). The capillaries were heated within the coiled filament of a Narishige P22 microelectrode puller (Narishige, Cambridge, UK) and pulled to a fine tip. The upper part of the borosilicate capillary was exposed to a naked flame for a brief amount of time repeatedly, resulting in an angled pipette with a fine tip used to trap the glomeruli.

A single glomerulus was isolated from a population of glomeruli (with no capsule and with the afferent and efferent arterioles still attached) under the confocal microscope according to successful labeling with R18 and AF488-BSA, and was secured in a perfusion bath by lowering the restraining pipette. After 5 minutes of further incubation with AF488-BSA, the glomerulus was imaged at 20X and recorded using the ND Acquisition video tool of NIS-Element AR (Nikon C1) software. The perfusion bath was then rapidly exchanged (≤1 minute) for an iso-oncotic fluorescence-free BSA. Glomeruli were scanned (1 frame every 4 seconds) for the duration of the experiment using a 488-nm wavelength laser, a 543.3-nm wavelength laser, and transmitted light. A pinhole limited the entrance of light to the photodetector. The imaging window was 1024 × 1024 pixels. For the whole experiment, the perfect focus system was left on to ensure acquisition of images with the same focal plane. These settings, including the gain, were kept the same for all experiments. Off-line analysis was performed using Nikon’s NIS Elements software. Only single capillaries, circular in cross-section and at the periphery of the glomerulus, containing labeled albumin, no red blood cells, and surrounded by a large pool of unlabeled albumin, were used for the analysis (Figure 1ci). The analysis was performed by drawing an ROI inside both loop and perfusion bath. ROI measurements of green fluorescent intensity mean versus time (seconds) were recorded from each experiment and plotted into a Cartesian coordinate system (Figure 1cii). The green line (Figure 1cii) demonstrates that the decline in fluorescence intensity follows a single exponential (R^2^ = 0.97). Of note we observed that the ROI within the glomerulus should be restricted to the capillary lumen and drawn without capillary wall interference. Ps’alb was then calculated from the rate of reduction of fluorescence intensity inside the capillary lumen using equations defined below.

### Calculation of Ps’alb values

The superficial glomerular capillary is treated as a well-stirred compartment from which the labeled albumin diffuses into the bath where its concentration is maintained at zero. The decay in fluorescence intensity is analyzed in a similar manner to that described by Daniels *et al.*^6^ From Fick’s first law of diffusion, the rate of diffusion of albumin molecules out of the capillary into the bath, -*dN/dt*, is proportional to the difference in concentration of labeled albumin molecules in the capillary and their concentration in the bath, (*C*_*C*_*-C*_*B*_), and the product of the permeability coefficient (*P*_*Salb*_) and the area of capillary wall through which diffusion occurs, (*S*), as follows:

(1) $$-\frac{dN}{dt}=P_{salb}\;S\left(C_{C}-C_{B}\right)=P_{Salb}S\left(\frac{N}{V_{c}}-C_{B}\right),$$

where *N* = the number of molecules in the capillary and *V*_*C*_ = the capillary volume so that *N/V*_*C*_ *= C*_*C*_. The negative sign indicates that labeled molecules are being lost from the capillary. If the concentration of labeled albumin in the bath is kept at 0, equation 1 reduces to the following:

(2) $$-\frac{dN}{dt}=P_{Salb}\cdot \frac{S}{V_{C}}\cdot N$$

Equation 2 can be immediately integrated to describe the loss of labeled albumin molecules with time as follows:

(3) $$N=N_{o}e^{-kt},$$

where *k = P*_*S’alb*_*S/V*_*C*_ and *N*_*0*_ = the number of labeled molecules at 0 time.

Because fluorescent intensity reflects the number of fluorescent albumin molecules in the region of interest (Figure 1d), equation 3 can be written as follows:

(4) $$If=if_{0}e^{-kt},$$

where If_0_ is fluorescent intensity at 0 time. The following expresses equation 4 as natural logarithms:

(5) $$\text{In}\left(If\right)=\text{In}\left(If_{0}\right)-kt$$

Thus, there will be a linear fall in ln(*If*) with time, and when plotted in Cartesian coordinates the negative slope of the relation is *k*. Providing the capillary is cylindrical in shape (and knowing the diameter of the capillary loop remains unchanged during the experiment, data not shown) the ratio of *S/V*_*C*_ is 2/R, where R is the capillary radius. *P*_*Salb*_ may then be calculated from *k,* because of the following:

(6) $$P_{Salb}=-kR/2$$

The minute following the wash period was the most appropriate to analyze (Figure 1g).

### Electron micrograph analysis

The Alcian blue fixed kidneys (and in some cases hearts) were processed for electron microscopy as previously described,^23^ imaged with a Technai 12 electron microscope (FEI, Hillsboro, Oregon), and analyzed with ImageJ (SciJava software ecosystem) under blinded conditions. The following were quantified on a grid section: eGLX depth, below 10 nm was considered “uncovered” and was expressed as a percentage of total measurements taken on a grid section; GBM length; podocyte and fenestration number per μm GBM; podocyte foot process width; and slit diaphragm width. GBM was measured using a grid tool for random sampling, and a minimum of 3 points were analyzed in each image (3 capillary loops per glomerulus and 2–3 glomeruli per animal) as previously described.^23^

### Diabetes model

Diabetes was induced in 6- to 8-week-old (200–380 g) adult male Sprague Dawley rats (Harlan Laboratories, UK) with a single dose of STZ, as previously described.^22^ Diabetes developed over 4 weeks, and glycemia, body weight, and urine were monitored or collected weekly. Glycemia was monitored via tail-tip blood droplets using an Accu-check (Aviva; Roche Diabetes Care, Burgess Hill, UK) glucometer. STZ-injected rats with glycemia <15 mM at week 1 were excluded from the analysis. Rat urine albumin was quantified using a rat albumin enzyme-linked immunosorbent assay (Nephrat; Exocell, Philadelphia, PA) according to the manufacturer’s instructions. Urine creatinine was quantified using an enzymatic assay (Thermo Fisher Scientific) on a Konelab Clinical Chemistry Analyser at the Langford Biochemistry Laboratory, University of Bristol, UK. At 4 weeks after injection (STZ or vehicle), glomerular albumin permeability was measured or rats were tail-vein injected with Ang1 (final blood concentration 200 ng/ml, corrected for body weight) or vehicle (0.1% BSA) for 30 minutes, as previously described for *in vivo* treatment,^16^ then terminally anesthetized. One kidney was perfusion-fixed via an abdominal aorta cannula, as described above for cardiac perfusion fixation in the mouse, and the tissue was processed for electron microscopy.

Rat paraffin-embedded kidney sections from nonperfused kidneys were stained using a periodic acid–Schiff kit according to the manufacturer’s instructions. Images were quantified with ImageJ using the H PAS color deconvolution plug-in. Corrected total cell intensity values were calculated as follows: intergraded density (area of glomerulus × background mean gray value). Ten glomeruli were analyzed per rat.

### Statistics

All statistical analyses were performed on a minimum of 3 separate replicates. Data are presented as means ± SEM. A *P* value <0.05 was considered statistically significant. For glomerular permeability experiments, *n* is indicated as follows: first number represents the number of animals and, in parentheses, the number of glomeruli analyzed for each group; that is, *n* = 3(9) indicates 9 glomeruli from 3 different animals. The number of animals was used for statistical analysis.

## Disclosure

MAS was on the advisory board for Retrophin and received consulting fees. All the other authors declared no competing interests.

## Supplementary Material

Supplementary Methods

Figure S1 Isolated glomeruli restrict the passage of high–molecular weight (HMW) proteins. (**A**) The relationship between HMW and low–molecular weight (LMW) dextran concentration and fluorescent intensity is shown (LMW R^2^ = 0.99, HMW R^2^ = 0.97). HMW (500 kDa) and LMW (4 kDa) fluorescein isothiocyanate (FITC)-conjugated dextran were perfused into mouse kidneys, and glomeruli were isolated. (**B**) apparent glomerular albumin permeability (Ps’alb) (permeability [cm/s]) was compared between FITC 500-kDa and FITC 4-kDa dextran (unpaired *t* test, number of animals indicated and number of glomeruli bracketed). Statistical analyses were performed on the number of animals. (**Ci**) Fluorescent decay of FITC-dextran (*n* = 7) was compared with fluorescent decay of AF488-dextran (*n* = 8) over 300 seconds and (**Cii**) quantified over a relevant time frame to analysis (100–120 seconds) (unpaired *t* test). ***P* < 0.001, ****P* < 0.0001.

Figure S2 The effect of perfusion with Ringer alone and of time on Ps’alb. The kidneys of (**A**) rats and (**B**) mice (lean db/+) were perfused with Ringer, followed in some cases by R18, then Alexa Fluor 488 bovine serum albumin (AF488-BSA; Ringer + fluorophores) and in other cases after Ringer perfusion (Ringer), the kidney was removed, and the glomeruli isolated and incubated first in R18 then AF488 to compare the rodent protocol with the human protocol (unpaired *t* tests). (**C**) Glomeruli from mice (1-year-old C57BL/6) isolated on the same day were left for 1, 2, 3, or 4 hours on ice before Ps’alb was measured (1-way analysis of variance). (**D**) Kidneys from the same mice were sieved on the day of use (same day) or left on ice in the refrigerator for 24 hours before being sieved (24 hours later) and glomerular Ps’alb compared (unpaired *t* test). ****P* < 0.001.
