# Supplementary material for: A novel assay provides sensitive measurement of physiologically relevant changes in albumin permeability in isolated human and rodent glomeruli
Source: Kidney Int. 2018 May;93(5):1086–97. doi: 10.1016/j.kint.2017.12.003 (PMC5912930; doi:10.1016/j.kint.2017.12.003)
Supplement: Supplementary Methods [file mmc1.docx]

**Supplementary material and methods:**

**Calculation of GSC values**

## *I-Calculation of GSC values considering that the glomerular membrane is penetrated by pores of constant radius:*

## According to the pore theory, if we think of glomerular filtration as occurring through a population of pores of uniform radii and assume that the pores are available to both water and albumin molecules, then we can define *Lp* as:

*Lp =* $\frac{A_{p}}{\Delta_{x}}\frac{R^{2}}{8\eta}$ (A1)

Where *A_p_/Δx* is the fractional unit area of membrane occupied by pores divided by pore length, *R* is the pore radius and *η* is the fluid viscosity. Similarly, the diffusional albumin permeability coefficient (*Ps’alb*) is expressed as:

*Ps’alb= Dϕf (a/R) (A_p_/Δx*) (A2)

Where *D* is free diffusion coefficient of albumin, *ϕ* the partition coefficient for the solute between the fluid within the pores and that in the solutions flanking the membranes and *f(a/R)* is a function of the solute’s molecular radius*, α*, to the pore radius describing the increased viscous drag on a molecule diffusing through a pore. These two functions*, ϕ* and *f(a/R)* are given by:

*ϕ = (1- a/R)^2^* (A.3a)

*f(a/R) = 1- 2.1 (a/R) + 2.09 (a/R)^3^-0.95(a/R)* (A.3b)

If *L_P_* is multiplied by *8ηD* and divided by Ps’alb, we are left with a ratio that is determined by the molecular radius*,* a, and the pore radius, R, i.e.:

$\frac{8\eta DL_{p}}{Ps'alb}= \frac{R^{2}}{\phi f(a/R)}$ (A.4)

The left-hand side (LHS) of the equation (A.4) can be evaluated for rat glomeruli using our experimental determined values for *Ps’alb* = 4.5 x 10^-7^ cm.s^-1^, *L_P_* = 1.629 x 10^-8^ cm^3.^ s^-1^ dyne^-1^ and taking *D*=9.23 x10^-7^cm^2^s^-1^, *η* = 0.676 x10^-2^dynes.s.cm^-2^. Substituting these values at the LHS of the formula we will have 177000x10^-14^ cm^2^ for the ratio, indicating a very high degree of restriction to movement of albumin through the pores. To estimate the right hand side (RHS) of equation (A.4), we have assumed a molecular weight for albumin of 67KD and used Venturoli and Rippe’s expression ^1^ to calculate a molecular radius of 3.522nm. To deal with the effects of the negative charge on the albumin, we used the empirical rule, which is to add 0.1nm to the molecular radius and subtract 0.1nm from the pore radius. We can argue that for evaluating the RHS side of equation A.4, one should apply this rule to the denominator only since that is the component of the expression which is affected. We then guessed values for R until one of these yielded a value for *R^2^/ϕ f(a/R)* that matched 177000x10^-14^. Using this restraint, a pore radius of 3.69nm gave a value for the ratio of 176700 x 10^-14^.

Having determined a value for pore radius, we estimated the reflection coefficient of the glomerular capillary using the expression:

*σ = (1- ϕ)^2^* (A.5)

When glomerular filtration rate is high, the sieving coefficient, θ (or GSC), has a value equal to *1-σ*. For a negatively charged albumin molecule being filtered through pores of 3.69 nm radius, this would mean that σ = 0.99932 and θ = 0.00068.

Pore theory is not robust enough to accept these values of *θ* that are based on a pore radius so close to the Stokes-Einstein radius of albumin, for it assumes perfectly spherical probe molecules and smooth surfaced passages cylindrical passages through the glomerular barrier and applies laws derived using continuum mechanics at a molecular level. Nevertheless the value reached for GSC (or θ) is very close to that arrived at from very different observations by Rippe's group.^2^

Because our values suggest such a high degree of restriction to albumin during ultrafiltration, a more reasonable approach is to follow Rippe and his colleagues^2^ and consider that albumin crosses the glomerular barrier through a very small number of “large” pores of radius equal to 11nm set in parallel to a large population of small, which albumin cannot penetrate.

## *II- Calculation of GSC values considering 2 populations of pores.*

Since the albumin molecule carries a net negative charge and we know the ratio *a/R,* with this value, we can estimate the partition coefficient and restriction factor for diffusion of albumin through the large pores *(ϕf(a/R)_L_).* This has a value of 0.1687. *Ap/Δx* for the large pores can then be estimated by substitution in a rearrangement of equation A.2:

$(A_{p}/\Delta_{x}){}_{L}{}=\frac{{Ps}^{'}alb}{D\phi f\left( \frac{a}{R} \right)}$ (A.6)

and with *Ps’alb*= 4*10^-7^cm/s and *D*= 9.23*10^-7^cm^2^/s, *(A_P_/Δx)_L_ =* 2.916cm^-1^. This value can now be used in equation A.1, to estimate the L_P_ of the large pores. With *R*=11nm, *Lp_L_* = 0.00652*10^-8^cm^3.^ s^-1^ dyne^-1^. At pores of radius =11nm, the reflection coefficient to albumin, *σ_L_*, can be estimated from equation A.5 as 0.3071.

The overall reflection coefficient of the glomerular membrane to albumin is determined by the sum of the products of the fraction of overall Lp and the reflection coefficient to the large pores and the large pores

*σ =* $\sigma_{L} \frac{(L_{P}){}_{L}{}}{L_{P}}+ \sigma_{S}\frac{(L_{P}){}_{S}{}}{L_{P}}$ (A.7)

Where *L_P_* and *σ* are mean values for glomerular membrane and *σ_L_*, *σ_S_* and *(L_P_)_L_*, *(L_P_)_S_* are values for the large pore and small pore populations respectively.

Taking the overall *Lp* of the glomerular membrane to 1.629*10^-8^ cm^3^.s^-1^ dyne^-1^, the large pores represent a tiny fraction of the overall *Lp* namely 0.004. Since the overall Lp is equal to sum of the *Lp* of the large pores and the small pores,

*(L_P_)_S_ = L_P_ - (L_P_)_L_*

Since *σs* = 1, substituting for *σ_S_* and *(L_P_)_S_* and rearranging equation A.7 leads to the overall reflection coefficient as:

$\sigma=1- \frac{\left( L_{P} \right){}_{L}{}}{L_{P}} (1-\sigma_{L})$ (A.8)

When equation A.8 is evaluated, σ has a value of 0.997228. Since *θ = 1- σ*, *θ* has value of 0.00277

While the exact values calculated using pore theory are open to criticism, they do indicate that the values for Ps’alb for rat glomerular capillaries reported in this manuscript and previously reported values of *Lp*, are consistent with a very high reflection and hence a very low value of θ of the glomerular capillaries that we have investigated.

The formula above was applied to human and mouse glomeruli using the values in table 1 to acquire GSC.

**References**

1. Venturoli, D, Rippe, B: Ficoll and dextran vs. globular proteins as probes for testing glomerular permselectivity: effects of molecular size, shape, charge, and deformability. *Am J Physiol Renal Physiol,* 288**:** F605-613, 2005.

2. Lund, U, Rippe, A, Venturoli, D, Tenstad, O, Grubb, A, Rippe, B: Glomerular filtration rate dependence of sieving of albumin and some neutral proteins in rat kidneys. *Am J Physiol Renal Physiol,* 284**:** F1226-1234, 2003.
