# Supplementary material for: A novel assay provides sensitive measurement of physiologically relevant changes in albumin permeability in isolated human and rodent glomeruli
Source: Kidney Int. 2018 May;93(5):1086–97. doi: 10.1016/j.kint.2017.12.003 (PMC5912930; doi:10.1016/j.kint.2017.12.003)
Supplement: Figure S1 — Isolated glomeruli restrict the passage of high–molecular weight (HMW) proteins. (A) The relationship between HMW and low–molecular weight (LMW) dextran concentration and fluorescent intensity is shown (LMW R2 = 0.99, HMW R2 = 0.97). HMW (500 kDa) and LMW (4 kDa) fluorescein isothiocyanate (FITC)-conjugated dextran were perfused into mouse kidneys, and glomeruli were isolated. (B) apparent glomerular albumin permeability (Ps’alb) (permeability [cm/s]) was compared between FITC 500-kDa and FITC 4-kDa dextran (unpaired t test, number of animals indicated and number of glomeruli bracketed). Statistical analyses were performed on the number of animals. (Ci) Fluorescent decay of FITC-dextran (n = 7) was compared with fluorescent decay of AF488-dextran (n = 8) over 300 seconds and (Cii) quantified over a relevant time frame to analysis (100–120 seconds) (unpaired t test). **P < 0.001, ***P < 0.0001. [file mmc2.pptx]

## Slide 1
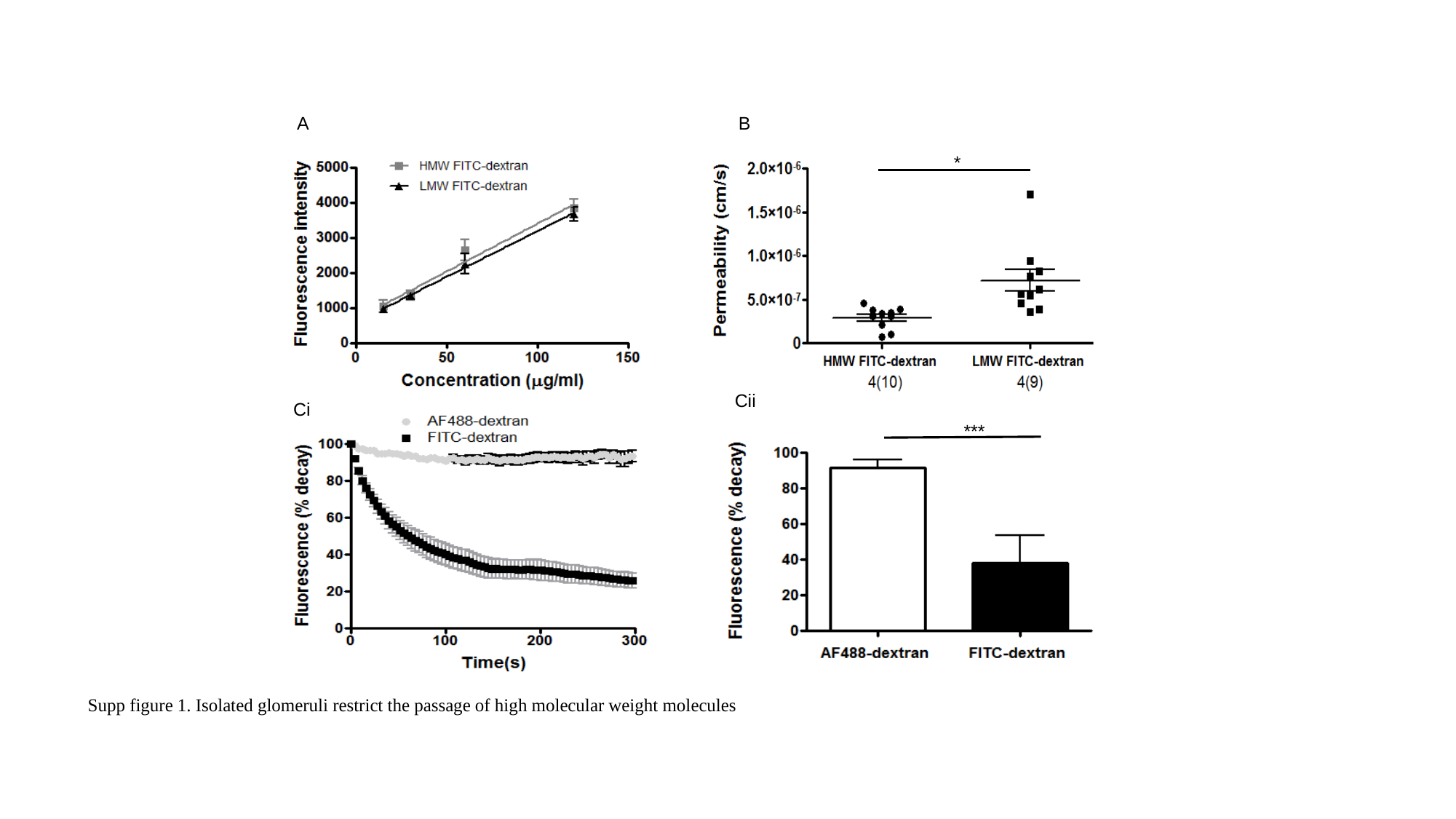

A
B
*
Cii
Ci
***
Supp figure 1. Isolated glomeruli restrict the passage of high molecular weight molecules
