# Supplementary material for: A novel assay provides sensitive measurement of physiologically relevant changes in albumin permeability in isolated human and rodent glomeruli
Source: Kidney Int. 2018 May;93(5):1086–97. doi: 10.1016/j.kint.2017.12.003 (PMC5912930; doi:10.1016/j.kint.2017.12.003)
Supplement: Figure S2 — The effect of perfusion with Ringer alone and of time on Ps’alb. The kidneys of (A) rats and (B) mice (lean db/+) were perfused with Ringer, followed in some cases by R18, then Alexa Fluor 488 bovine serum albumin (AF488-BSA; Ringer + fluorophores) and in other cases after Ringer perfusion (Ringer), the kidney was removed, and the glomeruli isolated and incubated first in R18 then AF488 to compare the rodent protocol with the human protocol (unpaired t tests). (C) Glomeruli from mice (1-year-old C57BL/6) isolated on the same day were left for 1, 2, 3, or 4 hours on ice before Ps’alb was measured (1-way analysis of variance). (D) Kidneys from the same mice were sieved on the day of use (same day) or left on ice in the refrigerator for 24 hours before being sieved (24 hours later) and glomerular Ps’alb compared (unpaired t test). ***P < 0.001. [file mmc3.pptx]

## Slide 1
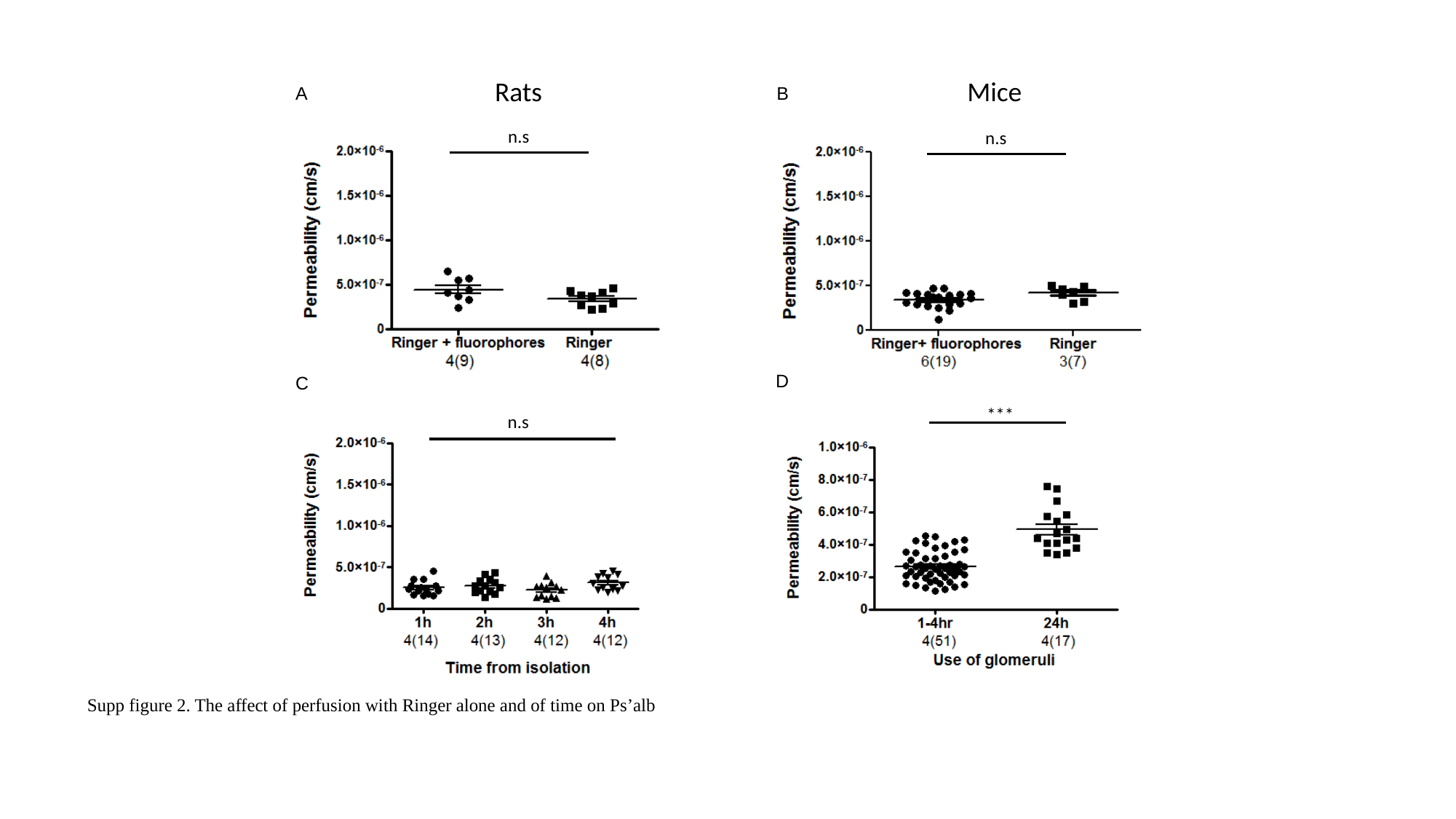

Rats
Mice
B
A
n.s
n.s
D
C
***
n.s
Supp figure 2. The affect of perfusion with Ringer alone and of time on Ps’alb
